# Professional quality of life and its associated factors among Vietnamese doctors and nurses

**DOI:** 10.1186/s12913-023-09908-4

**Published:** 2023-08-30

**Authors:** Anh N. P. Tran, Quyen G. To, Van-Anh N. Huynh, Khoi M. Le, Kien G. To

**Affiliations:** 1 Faculty of Public Health, University of Medicine and Pharmacy at Ho Chi Minh City, Ho Chi Minh City, 700000 Vietnam; 2 Laboratory Department, University Medical Center Ho Chi Minh City, Ho Chi Minh City, 700000 Vietnam; 3 Appleton Institute, Central Queensland University, 4701 Rockhampton, QLD Australia; 4 Science and Training Department, University Medical Center Ho Chi Minh City, Ho Chi Minh City, 700000 Vietnam

**Keywords:** Compassion fatigue, Compassion satisfaction, Burnout, Healthcare workers, Stress, ProQoL, Reliability, Validity

## Abstract

**Background:**

Prevalence of health workers with occupational health issues ranked fourth among all careers resulting in a reduction in quality of life. However, tools to measure professional quality of life (ProQoL) are unavailable in Vietnamese. This study aims to develop a Vietnamese version of the ProQoL, and examine ProQoL and its associated factors among doctors and nurses.

**Methods:**

The ProQoL is comprised of 30 items measures compassion satisfaction (CS), burnout (BO), and secondary traumatic stress (STS). The tool was translated into Vietnamese following the Guideline by Guillemin et. al (1993), reviewed by expert panels, and validated for internal consistency and test-retest reliability among 38 health workers working at hospitals in HCMC. The validated tool was then used in a cross-sectional study to measure the ProQoL of full-time doctors and nurses working in clinical departments at the University Medical Center, University of Medicine and Pharmacy at Ho Chi Minh City, Vietnam. In addition to the ProQoL, self-reported data about demographic and occupational characteristics were collected.

**Results:**

The Vietnamese version of ProQoL achieved high internal consistency (alphas between 0.85 and 0.91) and Intra-class Correlation Coefficients (ICCs between 0.71 and 0.89) for all subscales. Among 316 health workers, mean scores of CS, BO, STS were 36.4 (SD = 5.4), 24.9 (SD = 5.1), 25.9 (SD = 5.3), respectively, indicating moderate levels of CS, BO and STS. Participants who were older (b = 0.17, 95%CI = 0.08, 0.26), had sufficient perceived income (b = 2.59, 95%CI = 0.93, 4.24), and > 10 years of working experience (b = 2.15, 95%CI = 0.68, 3.62), had higher CS scores. Those who were older (b=-0.15, 95%CI=-0.23, -0.07), had sufficient perceived income (b=-2.64, 95%CI=-4.18, -1.09), > 10 years of experience (b=-1.38, 95%CI=-2.76, -0.01), worked in surgical department (b=-1.46, 95%CI=-2.54, -0.38) and 8 hours/day (b=-1.52, 95%CI=-2.61, -0.44), had lower BO scores. Moreover, those in a relationship (b=-2.27, 95%CI=-3.53, -1.01) and had sufficient perceived income (b=-1.98, 95%CI=-3.64, -0.32) had lower STS scores.

**Conclusions:**

The Vietnamese version of ProQoL is valid and reliable for use among Vietnamese health workers. Age, marital status, perceived income status, years of working experience, daily working hours, and specialty was associated with at least one component of ProQoL but gender, religion, education level, and monthly income were not.

## Background

World Health Organization estimates a worldwide shortage of 15 million healthcare workers by 2030, mostly in middle and low income countries such as Vietnam [1]. Healthcare workers are also at risk of exposing to biological, chemical, physical, ergonomic, and psychosocial hazards [2]. Prevalence of healthcare workers with occupational health issues ranked fourth among all careers [2]. Additionally, occupational stress was the highest ranked issue among healthcare workers which may result in a reduction in their quality of life [2].

The concept of Professional Quality of Life (ProQoL) which was firstly mentioned by Figley (1995) [3, 4] and then by Stamm (2010), [5]. is defined as “the quality one person feels and relation their work as a helper” (p. 8). ProQoL has two aspects which are compassion satisfaction (positive) and compassion fatigue (negative). Compassion satisfaction refers to the satisfying emotion obtained when helping others. Compassion fatigue refers to burnout and secondary traumatic stress because of regular exposure to other’s pain and suffering. Figley and Stamm also developed a scale to quantify ProQoL that have been validated and used in more than 26 languages [6]. However, the scale is not available in Vietnamese.

Previous studies found that compassion fatigue poses could have negative effects on both physical and mental health of healthcare workers including empathy decrease, drug and alcohol abuse, sleep disorder, lack of interest, and reduction in productivity and efficiency in patient care [7, 8]. In some cases, compassion fatigue leads to adverse mental disorders such as post-traumatic stress disorder, and depression [4, 9]. A meta-analysis of 71 studies worldwide showed that medical staff experienced burnout and secondary traumatic stress at a moderate level [10]. Another meta-analysis included studies between 2010 and 2019 showed that Asian health workers had the lowest compassion satisfaction but the highest compassion fatigue, compared to those in Europe and the U.S [11].

Factors associated with compassion satisfaction and fatigue have been investigated. Most studies found that being in the job for a long time and having a higher training helped medical staff manage stress better and improved compassion satisfaction [12–18]. Additionally, income and hospital benefit also influenced both dimensions of compassion fatigue [19]. In contrast, working long hours, more night shifts were positive correlation with exhaustion and burnout leading to increase compassion fatigue [14, 16]. Moreover, a meta-analysis found no association between age, gender, and marital status with both compassion fatigue and satisfaction [15]. While one study in Spain found that married participants had higher secondary traumatic stress scores than others, [20]. the scores were found higher in single/divorced participants in another study in China [17]. Associations between gender and compassion fatigue and satisfaction were also inconsistent with one study in Egypt found that female doctors was more likely to have burnout than male [14] which was in contrast to studies in Turkey [16] and China [17].

In Vietnam, fewer studies on quality of life were conducted among healthcare workers than other groups such as patients or the general population although studies on job satisfaction, [21] stress, [22] burnout, [23] quality of sleep [24] or anxiety and depression [25] were conducted. With a much smaller healthcare worker density than the world average (60 vs. 174/10,000 people), [26] there was a significant increase in the number of healthcare workers experiencing both mental and physical health problems, especially during the COVID-19 outbreak [27, 28]. Nevertheless, compassion fatigue and satisfaction in Vietnamese health workforce have not been well studied. Therefore, this study was conducted to develop a Vietnamese version of ProQoL scale; and examined ProQoL and factors associated with ProQoL of doctors and nurses at the University Medical Centre, University of Medicine and Pharmacy at Ho Chi Minh City (HCMC), Vietnam. The findings of this study help better understand about the ProQoL of medical professionals and assist with designing interventions to improve staff’s well-being and quality of service at the hospital.

## Methods

### Development of the Vietnamese scale

#### Description of the ProQoL

The Professional Quality of Life Scale (ProQoL) is comprised of 30 items which measure three scales: compassion satisfaction (10 items: item 3, 6, 12, 16, 18, 20, 22, 24, 27, 30), burnout (10 items: item 1, 4, 8, 10, 15, 17, 19, 21, 26, 29) and secondary traumatic stress (10 items: item 2, 5, 7, 9, 11, 13, 14, 23, 25, 28). The full description of these items can be found elsewhere [5, 6]. For each of the item, the respondent was asked to give a score from 1 “Never”, 2 “Rarely”, 3 “Sometimes, 4 “Often”, and 5 “Very Often” indicating the frequency of experiencing stated events in the last 30 days. Scores of each item were summed to create total scores for each scale. For the burnout scale, scores of 5 items (item 1, 4, 15, 17, 29) were reversed before calculating total score as instructed in the guidelines [5]. Levels of compassion satisfaction, burnout, and secondary traumatic stress were classified as Low (scores ≤ 22), Moderate (scores between 23 and 41), or High (scores ≥ 42).

#### Translation

This study followed the Guideline for Translation and Cultural Adaptation for Health-related Quality of Life Scale, which was developed by Guillemin et al (1993) [29]. After permission for using the ProQoL scale was obtained, it was translated into Vietnamese by two independent Vietnamese translators who are fluent in English. One translator worked in the health sector and was provided with information about the scale. The other worked in finance without knowledge of the scale. Discrepancies between the two Vietnamese versions were discussed among the research team members until consensus between the two was reached. This version was then translated back into English by another Vietnamese who are fluent in English and have been living in the U.S. for more than five years. This back-translated version was compared with the original version by a native English speaker living in the U.S. Any disagreement between the two versions was again discussed between the research team and the native English speaker, and adjustment was made accordingly.

#### Expert’s panel review

Two groups of participants were recruited to assess relevance and clarity of the Vietnamese scale. The first group including five experts with at least three years of experience working in this field. The second group included three doctors and three nurses regardless of their specialty. A semi-structured form was sent for the participants to rate each question for relevance and clarity on a scale of one (not relevant or clear) to four (relevant and clear). For ratings of two (need major revision) and three (need minor revision), the participants were asked to provide suggestions to improve the questions. There were also spaces in the form for the participants to provide comments as needed.

Content Validity Index, including Item-Content validity index (I-CVI) and Scale-Content Validity Index (S-CVI), was used to assess agreement among the participants [30, 31]. I-CVI was calculated as the number of participants with a rating ≥ 3 for each item divided by the number of all participants. S-CVI was an average of all items in the scale [32]. Additionally, Kappa coefficients taking into account agreement due to chance were also used. Questions with low I-CVI (≤ 0.79) [33] and Kappa (≤ 0.74) [30] were revised.

#### Evaluation of psychometrics

Psychometric characteristics of the final Vietnamese version was evaluated on doctors and nurses who worked full time in other hospitals in HCMC. These participants were recruited via email lists and personal contacts. Those who agreed to participate received information sheets and returned signed consent forms. The participants were then asked to complete a self-administered questionnaire at baseline and after two weeks. The period of two weeks was considered suitable as a trade-off between the changes in the underlying construct and biases by previous exposure to the questions. Internal consistency and test-retest reliability were evaluated.

### Main study

#### Study design and participants

This cross-sectional study was conducted at the University Medical Center (UMC) in June 2022. The UMC is one of the largest teaching hospitals in Ho Chi Minh City, Vietnam. With 1,000 beds and 3,300 staff, the UMC provides health care services to more than 2 million outpatients, 55,000 inpatients, and 30,000 surgeries per year. There are 34 clinical and 10 subclinical departments with a 30-bed ICU. Full-time doctors and nurses in clinical departments, who were directly involved in providing treatment and care to patients, were eligible to participate in the study. Similar to other studies, [34, 35] part-time staff whose characteristics are likely different from the full-time staff were excluded to reduce heterogeneity of the sample. A researcher met with eligible staff, provided information sheets, and invited them to participate in the study. Participation was voluntary. Staff who agreed to participate was asked to return signed consent forms. Convenient time when the participants were available was noted by the researcher. Meetings for data collection were then scheduled with the participants who were provided with a private space to complete a self-administered questionnaire.

#### Measures

Independent variables included demographic and occupational characteristics that were self-reported. Participants’ age was calculated by subtracting year of birth from the year of 2022. Gender was either male or female. Participants’ religions, marital status, and education level were grouped into having a religion or not, being in a relationship or not, and having a postgraduate degree or not, respectively. Two income-related variables including monthly salary (≤ 20 or > 20 million VND) and perceived income status (not sufficient or sufficient) were also reported. Participants’ specialties were grouped into surgery or internal medicine/other specialties. Participants were also grouped into having ≤ 10 years or > 10 years of working experience, and 8 hours or > 8 hours of working time per day. Outcomes included compassion satisfaction, burnout, and secondary traumatic stress scores.

### Analysis

Data were entered using Epi-data and analyzed with the use of Stata v.16.0. Internal consistency reliability of the Vietnamese ProQoL scale was assessed using Cronbach’s Alpha [36]. An alpha of at least 0.70 was consider acceptable although a value higher than 0.90 may suggest the scale contains redundant items. Intra-class Correlation Coefficient (ICC) was used to assess test-retest reliability [37]. Single measure coefficients from two-way mixed effects models with absolute agreement were reported, and if < 0.40 was considered “poor”, 0.40–0.59 “fair”, 0.60–0.74 “good”, and ≥ 0.75 “excellent” [37, 38].

For the main study, descriptive statistics including frequencies and percentages were generated for categorical variables and means with standard deviations were for continuous variables. Average compassion satisfaction, burnout, and secondary traumatic stress scores were presented for each demographic and occupational characteristics. Linear regression models were used to examine the associations between these factors with the outcomes, i.e., compassion satisfaction, burnout, and secondary traumatic stress. Model 1 was bivariate models that included each of the demographic and occupational factors as an independent variable and one of the three above outcomes. Model 2 was multivariable models including gender, religion, marital status, education level, monthly income, perceived income status, specialty, working experience, and average daily working time as independent variables and one of the three above outcomes. Age was not included in multivariable models due to its highly correlated with working experience. Regression coefficients and 95% confidence intervals were presented across all factors and outcomes. A significance threshold of 0.05 was used.

## Results

### Development of the Vietnamese scale

The Vietnamese version of the ProQoL scale was assessed for relevance and clarity by two committees. For the expert committee, the I-CVI was between 0.6 and 1.0 for relevance and between 0.8 and 1.0 for clarity; the S-CVI was 0.94 for compassion satisfaction, 0.88 for burnout, and 0.96 for secondary traumatic stress; Kappa for each item was between 0.42 and 1.00 for relevance and between 0.76 and 1.00 for clarity in the first round. Some words including “on edge”, “intrusive thoughts”, and “bogged down” were challenging to translate. Based on the experts’ comments, these items were revised to make it more culturally appropriate. Definitions for compassion fatigue and compassion satisfaction were also added at the beginning of the questionnaire to clarify these terms. After revision, all experts agreed that the items could now be used. Additionally, the second committee including doctors and nurses found the items relevant and clear.

A total of 40 participants was recruited for evaluating psychometrics of the Vietnamese scale. As two participants dropped out, test-retest data from 38 (22 doctors and 16 nurses) were used (Table 1). Internal consistency reliability calculated at baseline and after two weeks was high for compassion satisfaction (Cronbach’s alpha = 0.91 and 0.90), burnout (alpha = 0.86 and 0.88), and secondary traumatic stress (alpha = 0.86 and 0.85). Test-retest reliability was also good for compassion satisfaction (ICC = 0.88, 95%CI = 0.78, 0.94), burnout (ICC = 0.89, 95%CI = 0.79, 0.94), and secondary traumatic stress (ICC = 0.71, 95%CI = 0.51, 0.84).

**Table 1:** Internal consistency and Intra-class Correlation Coefficients for the scales

	Cronbach’s alpha at baseline	Cronbach’s alpha after two weeks	ICC (95% CI)
Compassion satisfaction	0.91	0.90	0.88 (0.78, 0.94)
Burnout	0.86	0.88	0.89 (0.79, 0.94)
Secondary traumatic stress	0.86	0.85	0.71 (0.51, 0.84)

### Main study

The sample included 87 doctors (27.5%) and 229 nurses (72.5%). The average age of participants was 31.9 (SD = 6.7) ranging between 23 and 55 years. The percentage of female health workers was 73.4% (Table 2). Most participants had an education level of below postgraduate (77.5%), ≤ 10 years of experience (74.4%), and perceived income status as sufficient/more than sufficient (85.4%). A majority of participants had a religion (61.7%), a monthly salary of ≤ 20 million VND (about USD800) (64.2%), and average daily working time of more than 8 hours (57.3%). Average scores for compassion satisfaction, burnout, and secondary traumatic stress were 36.4 (SD = 5.4), 24.9 (SD = 5.1), and 25.9 (SD = 5.3), respectively.

**Table 2:** Average scores (standard deviations) for each scale by sample characteristics

	n	Compassion satisfaction	Burnout	Secondary traumatic stress
All	316 (100.0)	36.4 (5.4)	24.9 (5.1)	25.9 (5.3)
Gender				
Male	84 (26.6)	36.6 (5.9)	25.1 (5.3)	26.2 (5.8)
Female	232 (73.4)	36.3 (5.2)	24.9 (5.0)	25.8 (5.2)
Religion				
No	195 (61.7)	36.0 (5.6)	25.1 (5.1)	25.5 (5.3)
Yes	121 (38.3)	37.0 (5.0)	24.7 (5.0)	26.5 (5.3)
Marital status				
Not in a relationship	158 (50.0)	36.2 (5.4)	25.7 (5.2)	26.9 (5.2)
In a relationship	158 (50.0)	36.6 (5.4)	24.2 (4.8)	24.8 (5.3)
Education level				
Below postgraduate	245 (77.5)	36.0 (5.3)	25.1 (4.9)	26.0 (5.3)
Postgraduate	71 (22.5)	37.6 (5.6)	24.5 (5.7)	25.6 (5.5)
Monthly Salary (VND)				
≤ 20 million	203 (64.2)	35.7 (5.5)	25.3 (5.1)	25.8 (5.4)
> 20 million	113 (35.8)	37.6 (4.9)	24.3 (4.9)	26.0 (5.2)
Perceived income status				
Sufficient/more than sufficient	270 (85.4)	36.8 (5.1)	24.5 (4.9)	25.6 (5.1)
Not sufficient	46 (14.6)	33.8 (6.1)	27.5 (5.1)	27.7 (6.3)
Specialties				
Internal/Others	173 (54.8)	36.6 (5.1)	25.5 (5.1)	26.4 (5.3)
Surgical	143 (45.2)	36.2 (5.7)	24.2 (4.9)	25.2 (5.3)
Experience (years)				
≤ 10 years	235 (74.4)	35.7 (5.4)	25.5 (5.2)	26.0 (5.4)
> 10 years	81 (25.6)	38.3 (4.8)	23.4 (4.5)	25.5 (5.1)
Average daily working time				
8 hours	135 (42.7)	37.0 (5.0)	24.0 (5.2)	25.5 (5.2)
> 8 hours	181 (57.3)	35.9 (5.6)	25.6 (4.9)	26.2 (5.4)

Table 3 shows factors associated with compassion satisfaction, burnout, and secondary traumatic stress. On average, for every year increase in age, the compassion satisfaction score increased 0.17 points (95%CI = 0.08, 0.26) and the burnout score decreased 0.15 points (95%CI = -0.23, -0.07). Gender, having a religion, education level, and monthly salary level were not associated with any outcome. Those in a relationship on average had lower secondary traumatic stress scores (adjusted difference (adif) = -2.27, 95%CI = -3.53, -1.01). However, its association with compassion satisfaction and burnout was not significant after adjusting for other factors. Perceived income status was consistently associated with compassion satisfaction (adif = -2.59, 95%CI = -4.24, -0.93), burnout (adif = 2.64, 95%CI = 1.09, 4.18), and secondary traumatic stress (adif = 1.98, 95%CI = 0.32, 3.64). The type of departments (i.e., surgical vs. internal/others) where the participants worked was not associated with compassion satisfaction or secondary traumatic stress but was associated with burnout with those working in the surgical department having lower burnout scores (adif = -1.46, 95%CI = -2.54, -0.38). Those having working experience of more than 10 years had higher compassion satisfaction scores (adif = 2.15, 95%CI = 0.68, 3.62) and lower burnout scores (adif = -1.38, 95%CI = -2.76, -0.01) than those having less working experience; the association with secondary traumatic stress, however, was not significant. Additionally, those with an average daily working time of > 8 hours had higher burnout scores (adif = 1.52, 95%CI = 0.44, 2.61) compared to those working an average of 8 hours per day; the associations with compassion satisfaction and secondary traumatic stress were not significant.

**Table 3:** Factors associated with each scale (regression coefficients and 95% confidence interval)

	Compassion satisfaction		Burnout		Secondary traumatic stress	
	Model 1	Model 2	Model 1	Model 2	Model 1	Model 2
Age (years)	0.17^***^ (0.08, 0.26)	n/a	-0.15^***^ (-0.23, -0.07)	n/a	-0.06 (-0.15, 0.03)	n/a
Female vs. Male	-0.25 (-1.60,1.10)	-0.01 (-1.44, 1.42)	-0.21 (-1.48, 1.06)	-0.06 (-1.39, 1.28)	-0.39 (-1.73, 0.95)	-0.11 (-1.55, 1.32)
Religion (No vs Yes)	1.08 (-0.15, 2.30)	0.95 (-0.24, 2.15)	-0.47 (-1.62, 0.68)	-0.47 (-1.59, 0.64)	1.02 (-0.19, 2.23)	1.01 (-0.18, 2.21)
In a relationship (Yes vs. No)	0.46 (-0.74, 1.65)	-0.75 (-2.01, 0.50)	-1.49^**^ (-2.60, -0.38)	-0.76 (-1.93, 0.41)	-2.10^***^ (-0.33, -0.93)	-2.27^***^ (-3.53, -1.01)
Postgraduate vs. Below postgraduate	1.63^*^ (0.21, 3.05)	0.78 (-0.93, 2.48)	-0.51 (-1.85, 0.84)	0.00 (-1.59, 1.59)	-0.38 (-1.79, 1.04)	-0.88 (-2.59, 0.83)
Monthly Salary (> 20 vs. ≤20 million VND)	1.88^**^ (0.65, 3.11)	0.93 (-0.62, 2.48)	-1.04 (-2.21, 0.12)	-0.32 (-1.76, 1.13)	0.22 (-1.01, 1.46)	1.49 (-0.06, 3.04)
Perceived income status (Not sufficient vs. sufficient)	-3.07^***^ (-4.73, -1.42)	-2.59^**^ (-4.24, -0.93)	3.02^***^ (1.46, 4.57)	2.64^***^ (1.09, 4.18)	2.10^*^ (0.43, 3.76)	1.98^**^ (0.32, 3.64)
Specialty (Surgical vs. Internal/others)	-0.37 (-1.57, 0.83)	-0.05 (-1.21, 1.11)	-1.33^*^ (-2.44, -0.21)	-1.46^**^ (-2.54, -0.38)	-1.18 (-2.36, 0.01)	-1.03 (-2.19, 0.13)
Working experience (> 10 vs. ≤10 years)	2.57^***^ (1.23, 3.91)	2.15^**^ (0.68, 3.62)	-2.03^**^ (-3.30, -0.77)	-1.38^*^ (-2.76, -0.01)	-0.54 (-1.90, 0.82)	-0.00 (-1.48, 1.48)
Average daily working time (> 8 vs. 8 hours)	-1.07 (-2.27, 0.13)	-0.96 (-2.12, 0.20)	1.65^**^ (0.53, 2.77)	1.52^**^ (0.44, 2.61)	0.65 (-0.55, 1.84)	0.57 (-0.60, 1.73)

*p<0.05, **P<0.01, ***p<0.001.

Model 1 was bivariate. Model 2 adjusted for gender, religion, marital status, education level, monthly income, perceived income status, specialty, working experience, and average daily working time

## Discussion

ProQoL known as a widely used scale to evaluate work-related compassion satisfaction and fatigue has shown its good validity and reliability across numerous studies [39, 40]. While the ProQoL has been translated into more than 26 languages such as Hungarian, [41] Greek, [42] Persian, [43] Japanese, [44] Arabic, Brazilian, Chinese, Danish, Dutch, Farsi, German, Korean, Polish, and Russian, [40] it was not available in Vietnamese. Following the standard translating and cultural adapting procedures, the Vietnamese version of the ProQoL scale was successfully validated. The Vietnamese version had high internal consistency reliability for all subscales which were comparable to the original scale [5] and those in Japanese, [44] Chinese, [45] Greek, [42] Spanish and Portuguese [46]. Additionally, the Vietnamese version had good test-retest reliability for subscales comparable to other scales such as Greek, [47] Japanese, [44] and Persian versions [43].

The participants also had quality of life scores comparable to those in a meta-analysis in 11 countries (compassion satisfaction = 32.59 ± 7.12, burnout = 26.92 ± 6.00, and secondary traumatic stress = 25.97 ± 5.36 ), [11]. and Nepal (compassion satisfaction = 40.56 ± 5.50, burnout = 24.85 ± 4.74, and secondary traumatic stress = 27.70 ± 6.23), [48]. and the U.S (compassion satisfaction = 39.77 ± 6.32, burnout = 21.57 ± 5.44, and secondary traumatic stress = 23.66 ± 5.87) [35]. The level of burnout and secondary traumatic stress in this study was also similar to those in Iran, [49] the Philippines, [19] and another meta-analysis of 28 studies in 38 countries [10]. However, most workers in the University Medical Center (98.7%) had a moderate to high level of professional quality of life which was higher compared to another meta-analysis of 62 Asian studies [11]. The inconsistency among studies may be due to differences in population characteristics, cultural context, and health policies where the studies were conducted [10, 15, 50].

Our findings showed that perceived income status was significantly associated with all three outcomes whereas monthly income was not. Health workers with lower financial burden experienced low burnout and secondary traumatic stress and had more compassion satisfaction, which was consistent with a study conducted in Greece [51]. The differences in results between perceived income and monthly salary is likely because monthly income in Vietnam does not reflect whether a person and their family live financially comfortable. Living with and support seniors are highly expected in Vietnamese culture and thus, the number of dependents may be large. While freelancing and owning home business are popular in Vietnam, it may not be registered and managed by the government. Therefore, perceived income may be a better indicator of their financial status. The findings that older health workers and those with more working experience had significantly higher compassion satisfaction but lower burnout were similar to those in studies in Egypt [14], US [3], Nepal [48], UK [52], Iran [53] and Taiwan [54]. A few studies in Vietnam also found negative associations between age and burnout among health workers [15, 22]. One explanation could be that older and more experienced workers as a result of being exposed more to hardship and struggles that faced patients may become more adaptable and have been better trained to manage stress and burnout [48, 55]. Young and less experienced workers may not be mentally prepared to face extreme circumstances that patients suffer during daily work. However, the associations between age with burnout and secondary traumatic stress were not significant in some other studies [20, 49].

While marital status was not associated with compassion satisfaction and burnout, it was associated with secondary traumatic stress. The mean secondary traumatic stress score among workers who were in a relationship were higher than those not in any relationship which is similar to findings in Turkey, [16, 56] Taiwan, [54] and a burnout study in Vietnamese health workers [23]. One explanation could be that having a partner/spouse provided additional social support to cope with stress at work [54].

Working long hours constantly can have harmful effects on physical and mental health as well as contributing to forming unhealthy behaviors (e.g., smoking and alcohol consumption) [57, 58]. One particular study have shown that working more than 40 hours/week was significantly associated with burnout [57]. In this study, doctors and nurses working more than 8 hours/day had higher burnout scores compared to those working less hours/day which is consistent with studies in the U.S, [35] Iran, [53] and Vietnam [23]. This is a challenging issue to address given the lack of human resources in low-middle income countries and high expectation that medical professionals should be prepared to work long hours.

The finding also shows that working in Surgical departments was associated with less burnout compared to those working in Internal/Other departments (e.g., Emergency and Outpatient). This is similar to the finding from a meta-analysis that the prevalance of burnout in Internal departments (35.8%) was higher than that in Surgical departments (32.8%) [59]. Other studies also found that people working in intensive care units had high mean scores of burnout and secondary traumatic stress; [11, 56] as these workers often encounter patients’ traumatic and life-threatening events and had to make critical treatment decision [11]. Other departments regularly experience high level of compassion fatigue include Pallitative care, Emergency, Oncology, Pediatrics, and Obstetrics [14, 15, 50, 56, 60, 61]. However, associations between compassion satisfaction and compassion fatigue and specialties were not significant in a study by Shafei et al. (2018) [14]. The inconsistency could be due to differences in study populations and contexts where the study was conducted [10, 15, 50].

This study was one of the first to investigate compassion fatigue and satisfaction in Vietnam. The strength of this study includes the use of robust multi-stage procedures (involving experienced translators, multi-disciplinary experts, and target audiences) in translating and validating the Vietnamese version of the ProQoL. The tool is now available and could be used not only by researchers but also health workers and managers in hospitals as a periodical check to detect compassion fatigue timely, and thus having early interventions to enhance work-related well-beings of health workforce, contributing to maintaining sustainable healthcare system in Vietnam. However, the study has some limitations. First, while the tool development involved multi-disciplinary experts and audiences from different settings, the main study was conducted in only one large hospital. Therefore, the result may not be generalizable to populations with other characteristics. Second, as with other self-reported tools, recall bias could occur although the tool has been validated with high validity and reliability. Finally, inference about causal relationship is not possible with a cross-sectional study.

## Conclusion

The Vietnamese version of the ProQoL is valid and reliable for use. Age, marital status, perceived income status, years of working experience, daily working hours, and specialty (surgical vs. internal/others) was associated with at least one component of ProQoL (i.e., compassion satisfaction, burnout, and secondary traumatic stress). Gender, religion, education level, and monthly income were not significantly associated with ProQoL.

This study implies that more supports are needed for younger and less experienced health workers to improve their professional quality of life. Providing advice on self-care (e.g., engaging in physical activities, having proper diet, and meditate regularly) may help recover after compassion fatigue [19, 50]. Having supportive programs (e.g. regular health screening, stress management and emotion control courses) and creating a healthy working environment can increase professional quality of life among health workers, increase their productivity at work, and ultimately improve patient care quality.

## Data Availability

The datasets generated and/or analysed during the current study are not publicly available due to the hospital policy but are available from the corresponding author on reasonable request.

## List of Abbreviations

Adif: Adjusted difference
I-CVI: Item-Content validity index
ICC: Intra-class Correlation Coefficient
HCMC: Ho Chi Minh City
ProQoL: Professional Quality of Life
SD: Standard Deviation
